# Association between urinary cadmium levels and prevalence of coronary artery disease: NHANES cross-sectional study (2009–2018)

**DOI:** 10.3389/fcvm.2024.1415269

**Published:** 2024-11-28

**Authors:** Xuhui Ma, Yang Yang, Qingjun Jiang

**Affiliations:** ^1^ Department of Cardiology, Hangzhou Xixi Hospital, Hangzhou Sixth People's Hospital, Hangzhou Xixi Hospital Affiliated to Zhejiang Chinese Medical University, Hangzhou, Zhejiang, China; ^2^ Department of Otolaryngology, Head and Neck Surgery, General Hospital of Ningxia Medical University, Yinchuan, Ningxia Hui Autonomous Region, China; ^3^ Department of Cardiovascular Medicine, People’s Hospital of Ningxia Hui Autonomous Region, Yinchuan, Ningxia Hui Autonomous Region, China

**Keywords:** coronary heart disease, urinary cadmium, metals, NHANES, cross-sectional study

## Abstract

**Objective:**

This study aims to sheds light on the correlation between urinary cadmium (Cd-U) exposure and coronary heart disease (CHD) and the mediating effects of serum alkaline phosphatase (ALP) based on a sample of adults in the United States.

**Methods:**

A comprehensive cross-sectional study was performed on 8,998 CHD participants who participated in the National Health and Nutrition Examination Survey (NHANES) from 2009 to 2018. Weighted logistic regression was employed to elucidate the association between Cd-U and the likelihood of CHD. We investigated the link of Cd-U exposure to the prevalence of CHD using limited cubic spline models to analyze the exposure-dose relationship. In addition, mediation analyses were conducted to explore the role of serum ALP in metal exposure-induced CHD.

**Results:**

8,998 participants were included, and 323 among them were diagnosed with CHD. Our study found that elevated levels of Cd-U in U.S. are linked to a heightened likelihood of CHD. Additionally, there is a non-linear positive correlation between Cd-U and CHD, and a saturation effect was observed. Further mediation analysis revealed that the association between Cd-U and CHD prevalence was mediated through serum ALP mellitus, with the mediation percentage being 2.5% (*P value* <0.05).

**Conclusions:**

Our study indicates a strong association between the levels of Cd-U exposure in urine and the likelihood of CHD, with serum ALP serving as a mediator.

## 1 Introduction

Coronary heart disease (CHD), the most prevalent form of cardiovascular disease, affects an estimated 20.5 million individuals aged 20 and above (1), and is the main cause of death and impairment in the developed world (2). CHD symptoms encompass angina pectoris, myocardial infarction (MI), and silent myocardial ischemia, whereas the development and progression of CHD are influenced by factors such as inflammation, hypertension, abnormal cholesterol levels, insulin resistance, environmental exposures, and infections (3, 4).

Cd is a toxic heavy metal that presents a significant threat to human health. It enters the body via air, water, soil and food. With a biological half-life of 10–30 years, it predominantly accumulates in many organs including the kidneys, liver, and bones, and damaging them irreversibly (5). Smoking is the principal source of Cd exposure. Inhaled tobacco, whether in the form of gas, particulate matter, or soluble chemicals, traverses biological barriers, enters the general circulation, and reaches a variety of target tissues (6). Furthermore, Cd has been proven to elevate the risk of cardiovascular disease through various mechanisms, including increasing blood lipid levels and inducing inflammatory responses (7). Cd exposure may also directly damage vascular endothelial cells, leading to endothelial dysfunction. This dysfunction can weaken the elasticity and regulatory capacity of blood vessels, and promote the progression to coronary artery disease, thereby leading to a higher risk of CHD (8).

Alkaline phosphatase (ALP) is a hydrolytic enzyme present in various tissues (9), and its activity in serum predominantly derives from liver and bones. Several studies have validated the effects of ALP on bone mineralization, as it promotes this process by regulating calcium and phosphorus metabolism (10, 11). Additionally, ALP has a crucial role in the liver and biliary system because it participates in the production and excretion of bile and aids in the digestion of fats (12). ALP is involved in vascular calcification, a key process in the pathophysiology of atherosclerosis, which is one of the main causes of CHD. Although ALP measurement is not typically used for the diagnosis of CHD, recent studies suggest that higher ALP activity may lead to an elevated likelihood of CHD (13). Plenty of studies have explored the link between Cd and cardiovascular disease (CVD), and indicated that Cd exposure, whether measured either in urine or whole blood, is correlated with a heightened risk of developing cardiovascular disease (14). However, epidemiologic studies linking Cd exposure to CHD prevalence are still limited. This study, based on data from the National Health and Nutrition Examination Survey (NHANES) conducted between 2009 and 2018, examined the connection between Cd-U levels with the rate of CHD in the US. The complex exposure-response relationships were characterized through multifactorial logistic regression. Investigating the influence of Cd-U on the occurrence of CHD could help identify modifiable environmental prevalence factors and guide the formulation of preventive measures.

## 2 Methodology

### 2.1 Data source

NHANES, a comprehensive nationwide cross-sectional survey, is accessible at (http://www.cdc.gov/nchs/nhanes.htm). The study protocol received approval from the Research Ethics Review Board of the National Centre for Health Statistics (NCHS). Each participant gave written consent during the recruitment process. The study utilized data from five cycles (2009–2018) of the NHANES database, which is maintained by the NCHS. The data were standardized and organized according to the recommendations of the NCHS through interview weights (https://wwwn.cdc.gov/nchs/NHANES/tutorials/default.aspx).

### 2.2 Population examination

**Figure 1:**
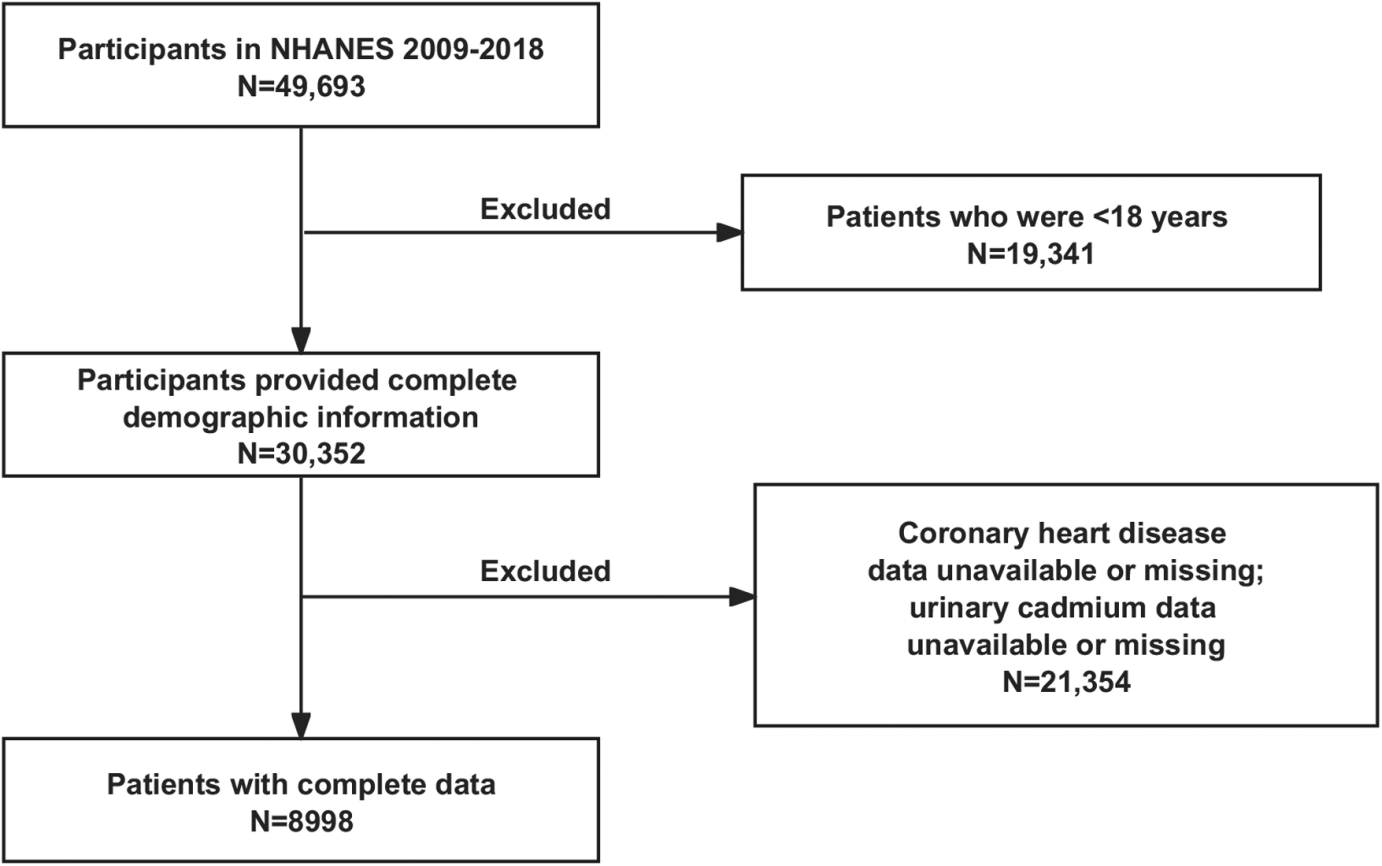
Flow chart of the current study. NHANES, National Health and Nutrition Examination Survey.

The NHANES project surveyed 49,693 individuals between 2009 and 2018. The study utilized rigorous inclusion and exclusion criteria to eliminate ineligible participants. The following were the exclusion criteria: (1) individuals under the age of eighteen; (2) individuals without demographic surveys; (3) individuals without urine metal testing; (4) individuals without CHD diagnostic data. Ultimately, 8,998 individuals were enrolled (Figure 1).

### 2.3 Data collection

Investigators receiving standardized training collected and documented all study data after rigorous interviews. The variables collected for the participant data included age, sex (female or male), ethnicity/race (Mexican American, non-Hispanic White, non-Hispanic Black, other race), marital status (married/cohabiting, never married or other: divorced/widowed/separated), educational attainment (lower or equal to high school: less than 11th grade education, high school graduation or equivalent; greater than high school: college or above), smoking (current smokers were defined as individuals who had consumed 100 or more cigarettes in the past and reported smoking on several days or every day; participants who had previously smoked fewer than 100 cigarettes but were not smoking at the present time were categorized as ex-smokers; those who had smoked fewer than 100 cigarettes in the past were categorized as non-smokers), drinking (non-drinkers were defined as never having had an alcoholic drink in the last 12 months; the rest were drinkers), poverty income ratio (PIR), and Body Mass Index (BMI) (it was calculated based on the formula: BMI = weight (kg)/height (m^2^)). The University of Minnesota Laboratory and the University of Missouri-Columbia examined additional biochemical measurements. Every experimental biochemical data set was examined to control the quality. The NHANES QA/QC processes met the standards established by the *Clinical Laboratory Improvement Amendments of 1988*. Of particular note, QA/QC guidelines are presented in the NHANES Laboratory Procedures Manual (LPM). Please visit (https://www.cdc.gov/nchs/nhis/tobacco/tobacco_glossary.htm) for detailed quality assurance and control procedures.

### 2.4 Metals measurements

The NHANES 2009–2018 study gathered data on Cd-U through an online solid-phase extraction technique in conjunction with tandem mass spectrometry and high-performance liquid chromatography. The objective was to detect the presence of Cd. The method enabled the detection of substances in urine samples with a concentration of 0.1–1.7 micrograms per liter (μg/L) based on isotope-labelled internal standards. It requires a minimum volume of 100 μl of urine sample. Urine samples were prepared, refrigerated in −20°C, and dispatched to the Division of Laboratory Sciences, National Center for Environmental Health (NCEH), Centers for Disease Control and Prevention (CDC), for analysis. The NHANES LPM provides comprehensive guidelines for both sample collection and sample processing.

### 2.5 Identification of CHD

The Medical Condition Questionnaire (MCQ) was used to establish the diagnosis of CHD. Participants who responded affirmatively to the question “Has your doctor ever informed you about CHD” were classified as having CHD.

### 2.6 Statistical analysis

The research was conducted with the help of R version 4.3.1, which is a freely available statistical software. The study also considered the intricate sampling design of NHANES. The demographic characteristics were evaluated through Chi-squared and *t*-tests according to the CHD status of patients. The link of Cd-U to CHD was analyzed through multivariate logistic regression. The analysis accounted for adjustments in gender, age, ethnicity/race, marital status, educational attainment, smoking and drinking, BMI and PIR.

In addition, we employed restricted cubic spline (RCS) plots to assess the association between Cd-U and CHD. This approach allowed us to better elucidate the correlation between Cd-U exposure and CHD prevalence. Subgroup logistic regression models helped to analyze the presence of specific groups in different subgroups and to test the stability of our results. We used the “mice” package to conduct multiple imputation analysis for participants data to address missing values and created five imputed datasets. Sensitivity analysis was also performed to detect noteworthy variations between the created dataset and the raw dataset. These investigations did not, however, reveal any noteworthy variations (Supplementary Table S1). We employed a two-tailed test for all tests, and deemed a *P* value <0.05 as statistically significant.

## 3 Results

### 3.1 Participant characteristics

8,998 individuals were encompassed in the study, with a weighted prevalence of 74,439,794. Out of the 8,998 participants, 323 developed CHD (Table 1). Furthermore, there were notable differences between those with CHD with respect to gender, age, ethnicity/race, education, marital status, PIR, smoking and drinking (*P* value <0.001).

**Table 1:** Baseline characteristics of participants with CHD, NHANES 2009-2018 (*N* = 8,998).

Variables	Level	Total, *N* = 8,998	NON-CHD, *N* = 8,675	CHD, *N* = 323	*P* values
Age, years (%)	<60	6,019 (74.1)	5,957 (75.6)	62 (27.9)	<0.001
	>60	2,979 (25.9)	2,718 (24.4)	261 (72.1)	
Gender (%)	Female	4,589 (51.3)	4,486 (51.9)	103 (35.6)	<0.001
	Male	4,409 (48.7)	4,189 (48.1)	220 (64.4)	
Marital status (%)	Married	5,359 (63.2)	5,170 (63.2)	189 (61.1)	<0.001
	Unmarried	1,694 (18.7)	1,675 (19.1)	19 (7.2)	
	Other	1,945 (18.2)	1,830 (17.7)	115 (31.7)	
Race/ethnicity (%)	White	3,501 (65.0)	3,298 (64.4)	203 (83.6)	<0.001
	Black	1,931 (11.2)	1,891 (11.4)	40 (5.1)	
	Mexican	1,324 (8.8)	1,300 (9.0)	24 (3.6)	
	Other	2,242 (15.0)	2,186 (15.2)	56 (7.8)	
Educational attainment (%)	<= high school	4,149 (38.2)	3,976 (37.9)	173 (47.1)	0.007
	>high school	4,849 (61.8)	4,699 (62.1)	150 (52.9)	
	<1.0	1,927 (14.8)	1,872 (14.9)	55 (11.2)	0.037
PIR (%)	1.0–3.0	3,852 (36.9)	3,693 (36.7)	159 (41.6)	
	>= 3.0	3,219 (48.3)	3,110 (48.4)	109 (47.2)	
	2009–2010	2,009 (19.4)	1,946 (19.5)	63 (15.9)	0.140
Year (%)	2011–2012	1,700 (19.3)	1,644 (19.3)	56 (16.9)	
	2013–2014	1,805 (20.1)	1,745 (20.2)	60 (18.2)	
	2015–2016	1,783 (20.4)	1,717 (20.4)	66 (20.2)	
	2017–2018	1,701 (20.8)	1,623 (20.5)	78 (28.8)	
BMI, kg/m^2^ (%)	<25	2,586 (29.3)	2,501 (29.4)	85 (25.2)	0.570
	>= 30	3,429 (38.3)	3,307 (38.2)	122 (43.2)	
	25–29	2,880 (32.4)	2,770 (32.4)	110 (31.6)	
Drinking (%)	No	1,666 (15.1)	1,563 (14.6)	103 (28.8)	<0.001
	Yes	7,332 (84.9)	7,112 (85.4)	220 (71.2)	
Smoking (%)	Current smoker	1,766 (18.8)	1,713 (19.0)	53 (13.4)	<0.001
	Former smoker	2,163 (25.1)	2,025 (24.5)	138 (43.8)	
	Never smoker	5,069 (56.1)	4,937 (56.5)	132 (42.9)	

Continuous variables are expressed as mean ± SD. Categorical variables are presented as *N* (%). NHANES, National Health and Nutrition Examination Survey; PIR, Income to Poverty Ratio; BMI, Body Mass Index; SE, Standard Error; n, number of subjects;%, weighted percentages.

### 3.2 Association between Cd-U with CHD

In order to elucidate the link of Cd-U concentration levels to CHD in adult individuals in the US, three logistic regression models were developed. The OR of the models can be interpreted as the probability of CHD with changes in Cd-U levels. Table 2 displays the correlation between Cd-U and CHD. In the unadjusted model, In the unadjusted model, a statistically significant positive correlation was observed between Cd-U and CHD (*P value <*0.0001). Importantly, this correlation remained statistically significant (*P* value = 0.014) even after adjustment for gender, age, race/ethnicity, educational attainment, PIR, and marital status in Model 2. Furthermore, in Model 3, by additionally adjusting the variables of smoking and drinking on the basis of Model 2, the association between ALP and CHD was still revealed to be statistically significant (*P *value = 0.006).

**Table 2:** Association of Cd-U with CHD patients in the NHANES 1999–2018 database (weighted) (*N* = 8,998).

Metal		Model 1		Model 2		Model 3	
		OR (95% CI)	*P* for trend	OR (95% CI)	*P* for trend	OR (95% CI)	*P* for trend
Cd, ug/L	Q1	1	*P* *<* 0.001	1	0.014	1	0.006
	Q2	1.93 (1.31–2.85)		1.45 (0.97–2.16)		1.47 (0.98–2.22)	
	Q3	2.37 (1.63–3.46)		1.48 (1.00–2.18)		1.52 (1.02–2.27)	
	Q4	3.03 (2.10–4.36)		1.62 (1.10–2.38)		1.65 (1.10–2.47)	

OR (95% CI) is used for urinary metals associated with CHD. Model 1 is unadjusted; Model 2 includes adjustment for gender, age, race/ethnicity, educational attainment, PIR, and marital status; In Model 3, gender, age, race/ethnicity, educational attainment, PIR, marital status BMI, drinking and smoking status were adjusted. Continuous metal variables is transformed; Q, quartiles.

### 3.3 Non-linear regression analysis of the correlation between Cd-U exposure and CHD

**Figure 2:**
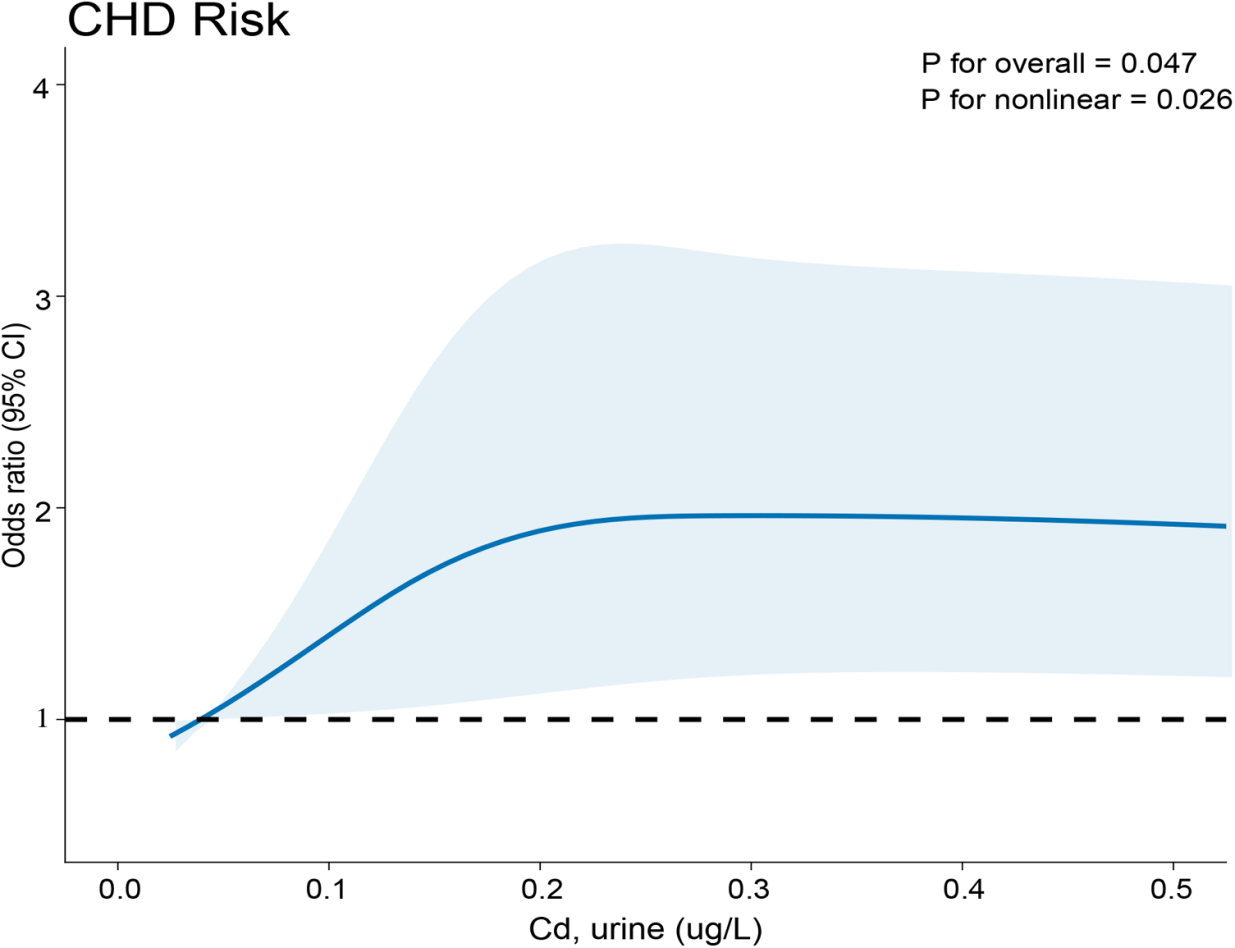
Relationship between Cd-U exposure and CHD. The *x*-axis indicates the concentration of metals in urine, while the *y*-axis reflects the likelihood of developing CHD.

This study utilized RCS plots as a visual tool to depict the connection between Cd-U levels and CHD prevalence. In this study, there was a direct correlation between Cd-U levels and CHD. A *p*-value of 0.026 indicated the linear relationship, and 0.047 shown the non-linear relationship. Furthermore, CHD prevalence increased with the Cd-U level (Figure 2).

### 3.4 Subgroup analyses

**Figure 3:**
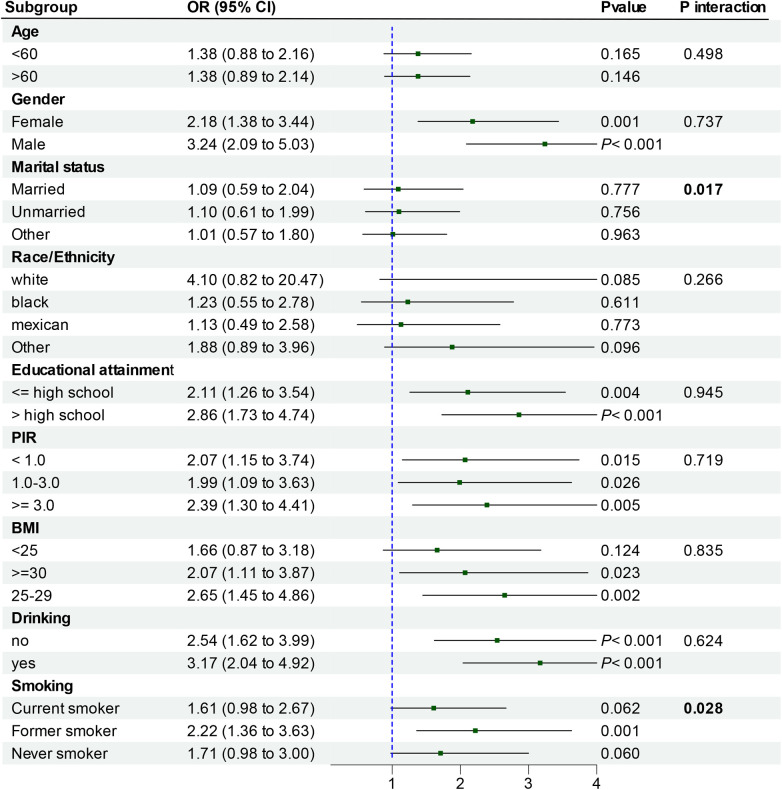
Subgroup analysis of Cd-U and CHD prevalence.

To facilitate result interpretation and identify potential sub-populations within the existing population, participants were grouped in terms of age, gender, ethnicity, education, PIR, BMI, smoking and alcohol consumption, and analyzed through categorical logistic regression (Figure 3). The correlation between Cd-U and CHD was noted in a subgroup comprising individuals aged over 60 years, males, those with higher educational attainment, PIR of 3.0 or higher, a BMI of 25–29, former smokers, and alcohol consumers (all *P*-values <0.05). Interaction tests revealed marked differences in the correlation between Cd-U and CHD in the married status and smoking strata. However, no notable differences was observed in the correlation between Cd-U and CHD with respect to age, gender, race/ethnicity, educational attainment, PIR, BMI and drinking. This indicates that factors other than marriage status and smoking did not significantly influence this positive correlation (*P value* >0.05).

### 3.5 Mediation analysis

**Figure 4:**
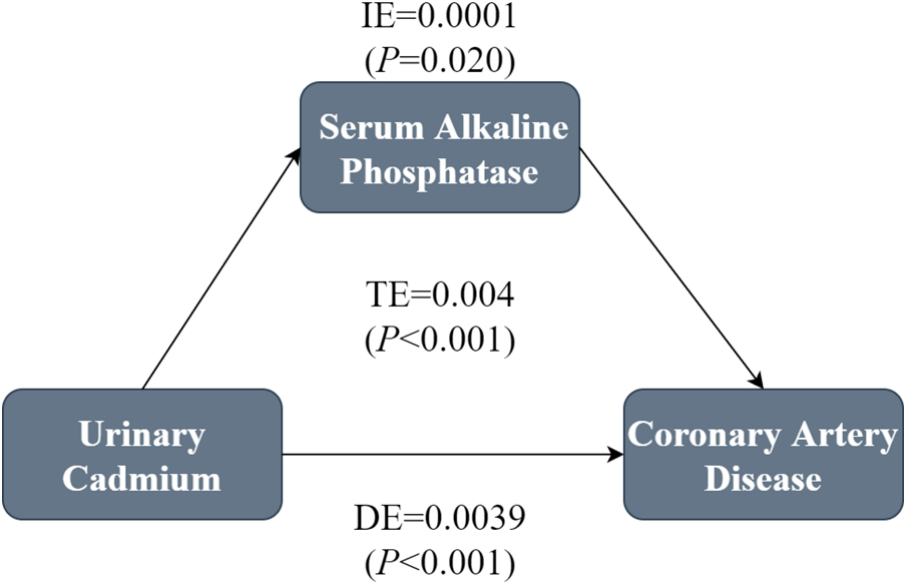
Mediation analysis of the association between Cd-U and CHD. In the mediation analysis, Cd-U was defined as the exposure factor; CHD was defined as the outcome; and serum ALP was defined as the mediating variable. Model was adjusted for gender, age, race/ethnicity, educational attainment, PIR, marital status BMI, drinking and smoking status.

A mediation analysis was performed to assess whether serum ALP mediated the correlation between Cd-U and CHD. The model and pathway for the mediation analysis are shown in Figure 4. After adjustment for all potential confounders, a marked indirect effect of Cd-U on serum ALP-induced CHD occurrence (*P* < 0.001) was observed, suggesting that serum ALP plays a partial role in mediating the effect. Despite the control of serum ALP, the influence of Cd-U on CHD remained statistically significant, which reveals direct and indirect effects. Nearly 2.5% of the effect of Cd-U on CHD was mediated by serum ALP.

## 4 Discussion

This study used data from five cycles (2009–2018) of the NHANES to assess the association between Cd-U and CHD. Higher levels of Cd-U exposure were found to be associated to a greater probability of CHD in adult patients residing in the United States. The correlation remained constant even in the fully adjusted model. Subgroup analyses and interaction tests proved that the positive link of Cd-U to CHD was more evident among those who were male, highly educated, former smokers and alcohol drinkers, with a PIR of 3.0 or higher and a BIM of 25–29. Interaction tests between subgroups showed significant differences between races, while other variables were relatively stable. RCS analyses revealed a nonlinear correlation between Cd-U and heart disease. Overall, Cd-U was positively linked to the development of CHD. It was also indicated that serum ALP mediated the relationship between Cd-U and CHD.

Cd exposure occurs through multiple pathways, including the ingestion of Cd-contaminated food and water, which constitutes a primary route of exposure (15). In addition, individuals may also be exposed to Cd through the inhalation of Cd-contaminated air and smoking (16). In the recent studies, Cd has been demonstrated to have a crucial role in smoking-induced cardiovascular disease. Our study proved a marked elevation in the correlation between Cd-U and CHD in former smokers. Furthermore, there has been a significant increase in the married population, a phenomenon that could potentially be influenced by the of secondhand smoke exposure (17, 18). Maternal exposure to high Cd concentration has been shown to greatly raise the likelihood of CHD in the offspring (19). Cd can affect the function of vascular endothelial cells through various mechanisms. Prolonged exposure to Cd can lead to endothelial dysfunction, which potentially leads to higher CHD risk (20, 21).

Elevated ALP level in serum serves as a common biochemical marker for multiple diseases affecting liver or bone, while its low level is an epiphenomenon of many severe acute injuries and diseases. Higher ALP level exhibited consistent links to all-cause mortality, cardiovascular mortality, as well as coronary artery calcification (22). Long-term exposure to Cd is positively correlated with liver damage. Cd can accumulate in the liver and damage liver cells, thereby impairing normal liver function (23). Since the liver is important in ALP synthesis and secretion, Cd -induced liver or bone metabolism disruptions may indirectly lead to changes in ALP levels. An increase in ALP levels may reflect liver-derived inflammation (19, 24). Increased C-reactive protein levels are related to higher ALP activity, and inflammation is a risk predictor for CHD (25). Therefore, changes in ALP levels may be a mediating factor through which cadmium exposure induces CHD via inflammatory pathways. In our study, serum ALP partially regulated the correlation between Cd-U and CHD. Although the mediating proportion was only 2.5%, the attention to ALP levels is equally crucial in the CHD.

Although the mechanism underlying the relationship between Cd and CHD mediated by ALP remains to be clarified, it is reasonable to attribute this phenomenon to cadmium exposure leading to liver damage and elevated ALP levels. The activation of Kupffer cells triggers cadmium-induced liver damage, and gives rise to the production of a large amount of pro-inflammatory cytokines. Kupffer cells release cytotoxic mediators, such as reactive oxygen species, reactive nitrogen species, bioactive lipids, as well as hydrolytic enzymes, which further exacerbate liver damage (26). Elevated ALP is an important indicator for confirming liver damage (27). It is noteworthy that smoking also causes elevated levels of ALP (28). Elevated ALP levels are often lead to a higher risk of atherosclerosis. ALP can catalyze the hydrolysis of inorganic pyrophosphate, an inhibitor of vascular calcification, result in vascular stiffening and promote the atherosclerosis process (29). The thickening and stiffening of arterial walls lead to reduced vascular elasticity and restricted blood flow. As the vessels gradually narrow, CHD, hemorrhage, and coagulation imbalances may be developed.

Nevertheless, our study does have several limitations. First, a causal relationship between Cd-U exposure and CHD can not be established because of its cross-sectional design. Furthermore, the variables associated with CHD were too intricate for us to consider all potential confounders, such as the metabolism vs. kinetics of heavy metals, that could have significantly influenced the results. Thirdly, NHANES did not include any clinicians who had received a professional diagnosis of CHD. Although the definition of CHD was contingent upon the participant's affirmation to the question, “Has your doctor informed you of your diagnosis of CHD?”, which is consistent with previous studies of CHD based on NHANES data, it is plausible that this approach introduces certain biases and the data were from NHANES are not indicative of the global population. Therefore, the findings need to be validated in other populations. Additionally, further prospective studies or randomized trials are needed to determine whether interventions with Cd-U can effectively prevent the occurrence of CHD.

## 5 Conclusion

In our study, the use of weighted logistic regression models demonstrated that Cd-U levels led to an increased prevalence of CHD. Nevertheless, further research is necessary to validate the correlation between Cd-U levels with CHD.

## Data Availability

The original contributions presented in the study are included in the article/Supplementary Material, further inquiries can be directed to the corresponding author.
