# Supplementary material for: Association between urinary cadmium levels and prevalence of coronary artery disease: NHANES cross-sectional study (2009–2018)
Source: Front Cardiovasc Med. 2024 Nov 28;11:1415269. doi: 10.3389/fcvm.2024.1415269 (PMC11634864; doi:10.3389/fcvm.2024.1415269)
Supplement: Supplementary file 1 [file Table1.docx]

Table S1 Association between Cd-U and CHD in the NHANES 1999-2018 database (weighted) before multiple interpolation

| Metals |  | Model1 | | Model2 | | Modl3 | |
| --- | --- | --- | --- | --- | --- | --- | --- |
|  |  | OR (95% CI) | P for trend | OR (95% CI) | P for trend | OR (95% CI) | P for trend |
| Cd | Q1 | 1 | *P <* 0.001 | 1 | 0.024 | 1 | 0.014 |
|  | Q2 | 1.93 (1.31-2.85) |  | 1.50 (0.98-2.28) |  | 1.55 (0.96-2.49) |  |
|  | Q3 | 2.37 (1.63-3.46) |  | 1.50 (0.99-2.26) |  | 1.63 (1.02-2.59) |  |
|  | Q4 | 3.03 (2.10-4.36) |  | 1.60 (1.06-2.40) |  | 1.71 (1.06-2.74) |  |

OR (95% CI) for urinary metals associated with CHD. Model 1 is unadjusted; model 2 adjusts the model for sex, age, race/ethnicity, education, PIR, marital status; model 3 adjusts the model for sex, age, race/ethnicity, education, PIR, marital status BMI, drinking and smoking status. Continuous metal variables is transformed; Q; quartiles.
